# The management of rare disease patients from a grassroot perspective: the role of patients’ organizations in the global recognition of rare diseases in Cameroon

**DOI:** 10.11604/pamj.2024.47.64.38226

**Published:** 2024-02-13

**Authors:** Rose-Danielle Ngoumou, Yves Bertrand Djouda Feudjio

**Affiliations:** 1 Department of Sociology, University of Yaounde I, Yaounde, Cameroon

**Keywords:** Rare disease, patient organization, grassroot perspective, Cameroon

## Abstract

**Introduction:**

rare diseases (RD) are extremely complex health conditions. Persons affected by these conditions in Cameroon are often neglected in society and health systems through the inexistence of policies and programs. In Cameroon, there exists no program or policy conceived to address their needs in terms of access to quality health care, timely and reliable diagnosis, treatments, education, etc. The consequence is that persons living with a RD (PLWRD) and their families do not participate in social life. The unique fate of PLWRD reveals that the principle of social justice and equity is flawed in Cameroon. However, patients, in order to survive in society, rely on patients’ organizations (PO) to improve their quality of life (QoL) and advocate for a better consideration in the society. The aim of this paper is to highlight how initiatives from a grassroot perspective like POs can inform decision-makers to address the needs of PLWRD and their families.

**Methods:**

the study associated a systematic literature review and semi-structured interviews with parents of children suffering from a RD and who are members of a PO. Through the systematic literature review we highlighted the impact POs have in the development of research on RDs, patient literacy, patient empowerment and advocacy while semi-structured interviews brought out the needs of patients and their families

**Results:**

findings, on the one hand show that, in Cameroon PLWRD face a number of challenges like the incurability of their condition, catastrophic medical expenses, stigmatization and marginalization, etc. and though in POs their QoL still remains poor. On the other hand, where POs are empowered they are key actors in research on RDs and help decision-makers on having a better insight into the type of RD that exists across a geographical area, the sociodemographic profile of patients, etc. for a better management of PLWRD.

**Conclusion:**

the study suggests that the ministry of public health should create a network with existing RD POs to adequately meet the needs of PLWRD.

## Introduction

In Cameroon, there is no official definition of rare diseases (RDs). According to EURODIS, a disease is rare when it affects 1 in 2000 persons. However, this definition varies from one country to another [1]. In the words of some authors: *« Rare diseases are chronic and serious, featuring early onset at birth or in childhood, rapid deterioration and high mortality rate, which creates a burden on society and public health systems »* [2]. In fact, children are the most likely to be affected by these diseases, as they *« are first manifest in the earliest years of life in more than 50% of cases and are responsible for more than 30% of infant mortalities »* [3]. Despite this situation, PLWRD in Cameroon are not always considered in the application of social and health policies. Patients and their families constitute a marginalized group. The challenges they face are numerous and they impose severe impacts on the family and society structure and hamper social development and individual fulfillment. *« People living with a rare disease are often at high risk of experiencing poor quality of life, including increased levels of anxiety, depression, pain, fatigue and limited ability to participate in society »* [4]. The impacts of RDs are felt right in the lives of the significant others of PLWRD. The first affected is the family, confronted with challenges that arise from the organization of the health system to the social organization of belief systems. Families can face constraints when accessing care, support, and assistance with their child´s atypical and unknown manifestations of disease. Medical inexperience and lack of knowledge on RDs remains the greatest challenge. There is also the problem of competing health priorities where priority is only given to diseases that have high prevalence like diabetes, cancer or HIV. It has been observed that: *« In low- and middle-income countries in Africa and Asia, health policy and planning towards universal health coverage is largely limited to high prevalence conditions to ensure the efficient expenditure of limited resources »* [5]. This low prevalence should however not justify that the health of PLWRD should be left to fate. Therefore, the authors add that: *« As these countries transition into middle- and higher income status, however, debates about allocation of resources will need to increasingly incorporate decisions about the management of less common diseases »* [6]. It is worth noting that, there is no clear information on the situation of RDs across the country, making it difficult for decision-makers to take action. In effect: *« it is often the very rarity of rare diseases that has left them largely disregarded by the research or medical community and policymakers »* [3]. Inspite of all this, there is a visibility of PLWRD in POs. In light with the aforementioned, the aim of this paper is to highlight the role POs can play in the recognition of RDs as a health priority in Cameroon.

## Methods

**Study design:** we associated a systematic scoping literature review and semi-structured interviews with parents of children suffering from a RD and who are member of a PO. Through the systematic literature review we highlighted the impact POs have in the development of research, patient literacy, patient empowerment and advocacy in order to give direction to relevant actions to improve the living conditions of RD patients in Cameroon while semi-structured interviews brought out the needs of patients and their families through the analysis of the challenges they face in society and the health care system. The systematic review was based on the question: « patients´ organizations and persons living with a rare disease in Cameroon » that was entered in research networks such as Google and Google Scholar. This research enabled us to obtain 90 articles containing data regarding the management of rare diseases at country and community levels, the role of patients´ organizations in the management of chronic diseases, patient advocacy. We then came up with a filter as well as inclusion and exclusion criteria of the articles to remain in the study objective. Following this exercise, 16 research articles were maintained definitely. Table 1 detailing this procedure can be viewed in the section reserved for tables.

**Table 1:** systematic literature review procedure

Database	Filter		Selected publications
	Date of publication	Variables (keywords)	
Google	1990-2015	Mothers of children with rare diseases, patients’ organizations	20
Google Scholar	2018-2023	Quality of life, rare diseases in health policies, children suffering from a rare diseases, rare disease management, rare disease treatments, cost of medical care, availability of information rare diseases, social support	70

**Study setting and population:** Cameroon is located in Central Africa with a variety of urban settings, including cities and towns. Its population was expected to reach 27, 224,262 by 2021. The study, however, used the populations of two urban settings within the country: Yaoundé in the center region and Douala in the Littoral region. Parents selected the setting for the semi-structured interviews, and interviews were conducted in private settings within the two aforementioned urban centers.

**Variables:** variables included cost of medical care, availability of information on the child´s condition, social support and participation in social life. Independent variables included catastrophic medical expenses, scarcity of information, lack of social support, stigma and labelling.

### Data collection resource and management

**Data collection tool:** we used an interview guide with open-ended questions for face-to-face interviews. We conducted in-depth, face-to-face interviews that were 45-60 minutes in length using open-ended and flexible questions with each parent. Topics included parents´ experiences with access to care in health facilities, social facilities for their children and their own social life.

**Data collection:** independent variables such as medical expenses, availability of information, social support, stigma and labelling were collected

**Sample size:** ten mothers were included in the study. Mothers were considered because in these patients´ organizations, mothers are more represented than fathers.

**Data analysis:** data collected from semi-structured interviews were submitted to content analysis. This allowed us to carry out a thematic analysis by bringing out the recurrent themes by grouping together all the answers relating to the same question by identifying and assembling similar recurring themes, which were summarized in their essentials, and counted in their occurrences for analysis.

**Ethical consideration:** the study was realized within the framework of a PhD research exploration, meaning the author obtained a signed research authorization from the head of department of the Sociology department of the University of Yaoundé I. This signed research authorization was then presented to the patients´ organizations we identified, we addressed a demand to collect data. We attached to the demand our research protocol, data collection tools, informed consent notice and information notice. The head of the patients´ associations after examining our request gave us a favorable notice. After shortlisting the parents, we were put in touch with those who agreed to participate in the study. Prior to commencing the semi-structured interviews, verbal and written informed consent was obtained from parents. Anonymity was provided for the parents.

## Results

In this part, we present the results we obtained from the analysis we realized. We start by analyzing the experience of mothers with a child suffering from a RD. This part focuses on the outcomes of semi-structured interview showing that mothers mainly face anxiety, stigma, financial hardship. The second part, drawing from the systematic literature review focuses on the role played by POs in creating visibility on RDs. We draw information from literature review and extrapolate with the experience of RD patients in Cameroon to point out the role existing patient organizations can play in favor of the recognition of RD patients and their families in the application of health and social policies.

**The experience of mothers:** persons living with rare diseases face numerous challenges that hamper their full participation in social life. Their condition attracts discrimination and stigmatization. persons living with a rare diseases represent a neglected population in society and mothers are the most affected as they become the primary caregivers of the sick child. The experience of mothers is difficult and results from catastrophic medical expenses, labelling and long-lasting stigma and difficult access to social life. Rare diseases in families is also the root cause of numerous violences perpetrated on women and children which need to be investigated to design preventive measures to these treatments. A recent study identified eight key needs of families who have a case of RD to handle. These were: *« 1) family-focused care, 2) Coping with uncertainty, 3) empathic communication, 4) practical support, 5) information, 6) psychological support, (7) interdisciplinary care, and (8) social support »* [7]. These findings corroborate with the result of the present study. In the interviews realized with mothers of children suffering from RDs, the challenges they enumerated were due to lack of information on the condition of their child, catastrophic medical expenses, lack of support and labelling and stigmatization.

**Lack of information:** misinformation and a scarcity of information on most RDs is a very serious problem faced by a lot of mothers who have to deal with a child affected by a RD. When the disease is identified, in most cases, mothers wait a long period before a diagnosis is made. Even when the disease is finally diagnosed there is usually little or no information on the precise treatments to offer as well as the physician that can adequately handle the rare condition. There is little or no health facilities that have the necessary resources (human, material and logistic) to handle RD patients. Also, in communities, families and society, these diseases are considered by a certain strata to be caused either by witchcraft or by divine retribution for a mother´s evil deeds. As a result of this, a therapeutic pattern is usually inexistent for these children and they are instead kept hidden in houses. A mother says: *« Till today I cannot tell you I know the specific doctor to see for my child. When she started manifesting symptoms I took her to a pediatrician, she told me she had Down syndrome (DS) but did not give me information on the causes of the condition, the treatment to give her or which specialist I had to go to. It is so annoying and exhausting »*M0. This is the plight of many mothers who have a child with a RD, they have to deal and handle a disease for which they have no clear information that can enable them take informed decisions on the health of their child. When it is not an absence of information, many parents go through the problem of misdiagnoses which in effect is the result of a scarcity of information on the disease. This is indeed a major setback for a lot of health professionals as they are not capable of adressing the needs of patients and their families causing them to go through psychological and physical pain. This is in line with Ni and Shi [2]. Who argue that: *« the lack of knowledge, misdiagnoses, missed diagnoses and incurability have posed formidable obstacles to physicians and patients in effective diagnosis and treatment »*.

**Catastrophic medical expenses:** the cost of medical care for a RD is not accessible to most parents. In the interviews we carried with mothers, we noticed that most of them cannot ascertain their monthly income as they have no stable professional occupation and as if that is not enough, they are usually the sole caregiver of the child. Even in the case where mothers have a regular source of income, they still face numerous financial challenges in accessing health care services for their children. This stems from the fact there is little expertise for most RDs and also limited infrastructures for these conditions. Moreover, health and care services are not subsidized for this category of patients. A mother reported that: *«The medications cost alot I assure you. I spend twenty-five thousand francs (25.000 FCFA) just for his medicines every month »*MO4. Out-of-pocket payments exclude alot of families from the health ecosystem as many have no consistent monthly revenue. In the same vein another mother says: *« I need money, I need support because I am single, I am a mother, I am a woman, I have to work. I do multi- tasking, when I get home now, I have to go to the market, I have to cook and children like him need special care, it takes alot of energy to cater for him. You see me thin like this, it´s because I do not eat well and because he exhausts me. In Europe such children have social security, their mothers are offered housing facilities. Everything is done for them, they go to school and their medical expenses are taken care of » MO2*. Mothers become physically and psychologically exhausted because as the needs of these children generate catastrophic medical expenses, they cause the impoverishment of the mother making her become economically, socially and emotionally vulnerable. Another mother says that if treatment for these conditions were subsidized it could help them handle the daily care and treatment of their child. This mother says: *« At least the government should make invalidity cards available for our children so that when we go to the hospital, the cost for treatment can be reduced, rare diseases are disabling conditions, in health centers and even in hospitals there is no reduction for our children. There is no reduction for medicines, you pay for medicines like everyone else. We really need help, if there was a reduction for the medicine he takes, that is, when you go to the pharmacy and present the child´s invalidity card so that a product that costs for example 8000 Frs or 10.000Frs the invalidity card can cover 5% or 10% of the price. It will really help us »MO3*. RDs being rare, industries and research agencies are not encouraged or motivated to invest in them as they see no benefit. In fact: *« Pharmaceutical companies are reluctant to invest in the development and clinical trials of drugs that will benefit a small market »* [8]. This indicates that there is a high risk for PLWRD to face financial hardships and end up not having access to basic health care.

**Stigma and long-lasting labelling: the cross of having a child with a rare diseases:** the incurability of most RDs affects the mental health of patients and their mothers as this becomes impossible for them to plan for the future. Having to live with a medical condition an entire life and for which there exists no cure is a bitter pill to swallow. In fact, the chronicity of a disease is always perceived in social representations as the cause of evil forces or witchcraft [8]. This causes the sick person to be stigmatized with their caregiver and to be viewed as persons of bad luck causing long lasting stigma on the child and also perpetuates more discrimination and isolation of mothers. These diseases deform the physical appearance of the child causing them to become different from the *« normal child »*. These can also give rise to various forms of violence that can go a long way to destabilize the family unit as well as the QoL of the caregiver. This calls for a greater empathy toward PLWRD and also for the design of interventions directed towards the improvement of the living conditions of this group of persons who though described as rare persons go through a lot of hardships that exacerbate them in the society. This seems important because: *« in rare diseases, the effectiveness of helping the patient has a broad impact. It translates into the quality of life of the patient's relatives, who are forced to “get sick” with him »* [9]. Relatives are forced to “get sick” and this includes physical exhaustion, emotional and psychological stress, stigma and self-stigmatization, etc. the stigmatization and social marginalization of both the child and their caregiver causes significant degradation of their QoL and health. Any child born with a RD is seen as a violation of traditional belief systems by the mother and causes her close relatives and family to suffer from ambivalent reactions. This has shown that stigma by association is common in daily interactions. The social identity of these women morphs as days go by because of the type of child they have. For DS the appearance of the child is not « conventional », it attracts confusion, stigmatization and wonder. These women are usually perceived as women who have committed a fault. When they occur, they are usually a first case a family experiences justifying why at times they are regarded as mystical. A parent of a child with hydrocephalus says: *« I was given derogatory names, I was called mama monster. The woman who gives birth to monsters » MO2*. Their social identity morph and becomes a painful experience to live.

**Difficult access to social life:** most PLWRD are excluded from social life because of their physical abnormality. Most of these conditions transforms the physical appearance of the patient and attracts stigmatization and rejection. In this study we found out from what mothers told us, that children suffering from RDs face an uphill battle accessing education and social life in general. An informant recalls how her son suffering from Down syndrome was refused admission into a public school. In her words she says: *« They are considered as mad persons. My son was refused admission into several schools. I was told since he wears diappers, he cannot be admitted and I should go with him to a specialized school for children with special needs. I told them I don´t have the means for such institutions but I still went there to find out. There, they told me since there is only one teacher and he wears diappers they cannot take him, stuffs like that » MO2*. These rejections makes families and patients to view the social sphere as hostile and makes them develop « survival instincts » that are not always helpful for the patient. In order, to prevent such attitudes towards these individuals, most families go into hiding with their child as a way of protecting them. Njelesani *et al*. in their study describe the same situation in these words: *« As a result of violence being all too common, some children with disabilities are kept at home and not attending school. Parents described the need to protect children by keeping them at home since schools are often perceived to be unsafe. In these cases, children with disabilities are not receiving the benefits of an inclusive educational setting such as increased academic achievement, peer acceptance, and self-esteem, a richer friendship network, and positive life time benefits (higher salaried jobs, independent living) »* [10]. This is what restricts the participation in social life of many children suffering from a RD. Their physical appearance discredits them and causes them to be stigmatized and rejected; their families progressively withdraw them from the social landscape to offer them protection. What might seem to these parents as a resilience strategy instead reduces this child´s chances of participating in social life to zero and this explains why most of these children have no basic formal education, no medical attention nor leisure time.

**The role of patients´ organizations in the visibility of rare diseases:** patient organizations occupy a very strategic place in the advocacy for RD patients. Their actions are anchored in the premises of leaving no one with a RD behind especially in this era where universal health coverage (UHC) is at the top of the agenda of universal health policies. Caring for a child suffering from a RD can weigh a heavy toll on the mother; this can have significant impacts on their mental health and QoL. The majority of “treatments” proposed to these children are usually palliative care treatments that only relieve symptoms. The mother has to develop strategies and techniques that can help sustain her QoL and that of the sick child. One of these strategies is adhering to support groups or POs. A number of authors argue that these organizations have the necessary ressources to impulse strategic interventions for PLWRD. The accumulated knowledge they have from the experience of each case they meet in their organization makes them key actors in paving the way for decision makers to take operational measures in adressing their specific needs. We identified a total number of seventy (70) articles published from 2018 to 2022 that focused analysis on research in the field of RDs, especially those that laid emphasis on the role of Pos in the development of « opportunities » for patients in terms of therapeutic innovations, the role of PO in mitigating the effect of disparities in accessing social and health facilities.

**Patients´ organizations as knowledge production and knowledge distribution in health governance:** a number of authors are now unanimous on the central role POs play in attracting significant interest on RDs in society. In fact, their actions, when well tailored are described as an « evidence-based activism » [11]. Rare diseasess are a global challenge for health systems as there is very little knowledge about them. Health professionals « miss cases » of RDs, because they are categorized as cultural and social diseases and managed accordingly. Rather than go to the hospital, most patients end up closing themselves in houses or finding support groups where they can open up about what they experience daily. This makes these POs to become strong channels of knowledge production and distribution because the condition for families and patients to join these organizations is that they should be capable of sharing their experience with living with a disease. This usually has to do with access to health care services, social infrastructures, treatment, etc. This is how POs accumulate information on specific diseases, that they can diffuse to inform the general population and decision makers. Indeed, as Marvis and Le Cam put it: *« the greatest barrier to the prevention, diagnosis and treatment of rare diseases has been and still is insufficient knowledge of the mechanisms and the natural history of the various diseases »* [12]. RDs are existing health events in Cameroon that are difficult to appreciate because of the limited information on these diseases. RDs are perceived negatively in many Cameroonian cultures. This is due to the high complexity and confusion they give rise. Patient organizations are therefore important platforms that generate evidence-based informations on the real aspects of RDs that are important to inform decision-makers.

**Advocacy and patient empowerment:** The role of POs is key in empowering patients so that they can take actions for their own health. Empowerment is a very important asset in enhancing health promotion. It is even more important in managing RDs in their complexity and addressing the needs of patients, therefore: *« empowerment is a necessity for patients with rare diseases, which are chronic, difficult to manage, so rare that coordinated efforts are imperative to make progress, and largely disregarded by the research or medical community and policy makers. It has been effectively applied in the rare diseases community, as exemplified by the role of patients´ organisations in fostering appropriate policy, research, and health care provision »* [13]. Vulgarizing knowledge on RDs is a key strategy in empowering patients and carrying advocacy as the scarcity of information concerning RDs puts patients and their families in a complexe situation of vulnerability. With this information , patients, over time, learn how to become knowledgeable about their condition and acquire skills that make them become patient experts. Patient organizations that are well organized often work in collaboration with organizations that diffuse information on RDs to their members making them capable of managing their health. Daban *et al*. in their study have showed how the French Chronic Myeloid Leukemia (CML) association played a vital role in informing and empowering patients on different treatments available regarding their condition. By making available drug sheets for patients, these helped patients gain more literacy on their disease and drug safety. Infact: *« these drug sheet included a summarized information on name (brand name and generic form if available), indication (first line or/and second line), date of approval, mechanisms of action, the main adverse effects, the drug-drug interactions and also the drug interaction with herbal products »* [14]. As a result of this, patients become experts in their own disease. They come together in associations and groups to diffuse their expertise to « new » patients and their families but also inform the health system on the real dynamics of the experience of the disease.

**Research actors:** The role of POs in research has become a topical issue in adressing the needs of PLWRD. Patient organizations live on a daily basis with patients and can best identify what their needs are. As patient representatives they can inform research industries on the areas that need investment in research and can also provide participants for clinical trials eventhough some authors suggest that the role of patients in research should be acknowledged beyond this aspect and *« they should not be considered only as subjects in clinical trials »* [13]. Instead: *« collaborations between the patient community and research sponsors must be guided by transparency, mutual interest, shared values, agreed codes of conduct, and ratified terms of engagement for PO and industry collaborations. The collaboration must be underpinned by the willingness of research sponsors to learn and optimise initiatives to meet patient and caregiver needs and expectations »* [15]. Many POs have limited resources, often relying on donations, families and local grassroot efforts to raise funds for support. This is how they end up forming advocacy blocs dedicated to educating, connecting and supporting all stakeholders, including other patients, advocates, caregivers, pharmaceutical industry, researchers, clinicians, and healthcare providers. Through these efforts, they help further disease research, obtain regulatory approval for new treatments and ensure access to care.

## Discussion

The results of this study which aimed to show the role of patients´ organization in the recognition of rare diseases in Cameroon. By pointing out the lived experience of mothers of children suffering from a RD, it reveals that globally parents face adverse effects of the implications of their children´s conditions. However, patients´ organization contribute in giving a hope to parents through advocacy, sensitization, research and patient empowerment. This is because POs realize that *« coordinated efforts are imperative to make progress »* [13]. In countries like Cameroon that do not have a regulatory framework for RDs nor a national registry [16], such initiatives are to be encouraged. Infact, it is through these organizations that patients and their families learn how to become knowledgeable about their condition and become experts in their own disease. This is due to the fact that there is limited knowledge of how to handle these diseases. Many healthcare practitioners remain confused in front of a situation of a RDs and this impacts the care and treatment of patients as an author remarks it that patients with RDs face: *« referral bias, generally meaning a lack of referral from general practitioners (family physicians) to specialist services, and the fact that many Indigenous people live in remote locations that can be hundreds and thousands of kilometres from where clinical genetics services are based, even with, sometimes geographically broad, outreach services »* [17]. This points out the difficult life of patients and families with the health care system. Meaning that more advocacy need to be carried by patients and their families represented by their support groups to make their voice heard and also to inform research and medical practitioners of their conditions

## Conclusion

This study allowed the researchers to uncover insights into the stigma experiences of ten mothers who have a child suffering from a RD in Cameroon and the role of POs in the global recognition of RDs. The study also highlighted the experience of families (mothers) handling a case of RD. The study showed it is emotionally and psychologically draining as these women find little or no support both from the society and their families. The psychosocial challenges faced by these mothers range from being exposed to stigma and discrimination, to dealing with the anger and fear of not being able to give adequate and long-lasting care to their children. In many cultures these children are regarded as a sign of ill omen, witchcraft, curse, misfortune, etc. The point is, these children often viewed as « snakes », « wizards » are treated accordingly. It is therefore a great relief when a parent can relate with other parents who deal with the same burden. This is why many prefer to liaise with support groups, where they can identify themselves to other persons who face the same daily hardships. This is how for most parents, joining a support group becomes a resilience system they put in place to « survive » the stress endured due to rejection from a spouse, family members, friends, etc. In this sense, the PO is very important in improving the living conditions of RD patients and drawing attention to their fate. This offers an ideal vantage point to identifying the role these organizations can play in the visibility of RDs. As Aymé *et al*. say: *« patients´ organizations have a role at all levels, from research funding to regulatory aspects of the orphan-drug market, including production of educational information, and the design of public policy and study projects. The notions of rare diseases and orphan drugs were established by a coalition spearheaded by patients´ organizations and included the academic community »* [13]. There are a number of existing patients´ organization in the country, but they are not always supported by the government in their activities. But not only that, these organizations possess enough information on patients that can inform decision makers on « unexplored zones » in RDs for appropriate action.

### What is known about this topic

- *Rare diseases are complex debilitating health conditions;*
- *Patients’ organizations are key actors in research on rare diseases*.

### What this study adds

- *This study explored the lived experience of mothers of children suffering from a rare disease both in the hospital setting and their families in Cameroon;*
- *The study has highlighted the role of patients’ organizations in data production to address the needs of PLWRD and their families;*
- *The study contributes to raising awareness on rare diseases in Cameroon*.
